# Grand Challenges in Synthetic Biology to be Accomplished

**DOI:** 10.3389/fbioe.2013.00002

**Published:** 2013-06-12

**Authors:** Pengcheng Fu

**Affiliations:** ^1^State Key Laboratory of Heavy Oil Processing, Faculty of Chemical Engineering, China University of PetroleumBeijing, China

When engineering science meets biological science, synthetic biology is created. Over the past half century, systems engineering has seen numerous successful applications in the engineering field such as manufacturing, electronics, telecommunications, computer, and networks, etc. At the same time, biological systems have been dealt in a reductionist way which resulted in accumulation of numerous but relatively fragmented biological information on genes and proteins, and their interactions. With the advent of genomics and other high-throughput technologies, biological paradigm has been shifted to a holistic view on a living system as a whole for understanding of complex life phenomena and living systems and for modification of genes, proteins, metabolites, and other cellular components in order to obtain novel functions/products.

As an emerging biological research field, synthetic biology has shown its potential in application of engineering formalisms to design and build functional modules from nucleic acid and protein “parts” and then to integrate such modules into an existing biological systems for novel functions, or to create novel life forms by reconstructing the cellular signaling, regulatory, and metabolic building blocks. The artificially re-designed biological systems may allow for experiments that would be too difficult, if not impossible, to be conducted in their full natural context. Although the ultimate goals are such as the creation of artificial life, so far applications of synthetic biology are typically demonstration of engineering concepts, systematic design, and module assembling in cellular systems (Purnick and Weiss, 2009). The resultant biological parts and modules been designed include switches (Gardner et al., 2000; Kramer et al., 2004), logic gates (Hooshangi et al., 2005), pulse generators (Basu et al., 2004), time-delayed circuits (Weber et al., 2007), oscillators (Stricker et al., 2008), sensors (Basu et al., 2005), and regulators (Zhang et al., 2012). These modules can be used to regulate gene expression, manipulate protein function, modify metabolism, and signal cell–cell communication. On the other hand, care must be taken that different from engineering systems, biological systems are capable of replication, extremely complex in their non-linear network and component interactions, and with less known mechanism for both their basic elements and the whole system. As we ponder the future directions in biology research that combines the exploratory nature of biology with the constructive nature of engineering (Purnick and Weiss, 2009), there remain many main scientific challenges in synthetic biology, some of which are as follows:

## 1 Modeling for Synthetic Biology Practice

Cells can be viewed as interwoven networks of interactions among proteins, DNA, and metabolites involved in signaling, material, and energy transfer. Understanding of cellular systems and then modification of them require quantitative models which are capable of predicting complex intracellular interactions of biological elements, their modules and networks. A whole cell computational model has been built up for this purpose that has used data from more than 900 scientific papers to account for every molecular interaction that takes place in the life cycle of *Mycoplasma genitalium* (Karr et al., 2012). This type of detailed models need to be expanded to allow simulation for synthetic biology applications, such as prediction of complex phenotypes for biological chassis bearing heterologous genetic parts, or compatibility of cellular networks and the incoming artificial modules.

## 2 Device Modularization and Standardization

So far many genetic device and small modules have been constructed and accumulated in the first wave of synthetic biology (Purnick and Weiss, 2009). These basic elements have been simple and are used to perform isolated cellular functions. In order to create larger functional architecture by synthetic biology using engineering design principles, modularization, and standardization become a necessity. However, the non-linear and uncertain nature of biological systems makes standardization of parts a non-trivial task. More efforts are needed to create general and stable artificial parts and modules which can be “plugged and played” by synthetic biologists in considering the characteristics of cellular systems, such as cell growth and differentiation, mutation, evolution, and other cellular activities.

## 3 Encoding Life with Non-Natural Codons

Biosynthesis of proteins with non-natural amino acids has drawn great interests with the prospects for novel, artificial genetic code which may ultimately lead to the creation of new life forms. Production of proteins and/or man-made polymers with non-natural amino and keto acids (Wang et al., 2003) has been achieved by forming an acylated transfer RNA chemically (Robertson et al., 1989; Noren et al., 1990), by use of modified aminoacyl tRNA synthetases (Chin et al., 2003; Mehl et al., 2003), or by producing new codons with four or five bases (Hohsaka et al., 2001a,b), or codons containing non-natural base pairs (Wang and Schultz, 2002). This technology need to be spurred by the goals of using existing cellular machinery to produce proteins with desirable new functions (Mehl et al., 2003) and to design codons and protein scaffolds to develop protein engineering for novel therapeutics (Nuttall and Walsh, 2008).

## 4 Quest from Minimum Genome

Synthetic biologists are interested in the quest for artificial cells, using either “top-down” or “bottom-up” approach which aims at determining the smallest set of genes, molecules, and structures for cell replication, growth, metabolism, and regulation that comprise life from two different aspects. By the top-down method, a minimal genome is generated by systematically reducing a cell's genome until it loses its functions (Glass et al., 2006). On the other hand, the bottom-up approach involves assembling the components or information units from scratch until an aspect of life emerges (Bedau, 2003). Study for such a minimal gene set and its features may shed light into the basics of cellular functions and help to determine the subset of essential genes in most species. Artificial cells allow combining properties of biological systems for therapeutic and diagnostic applications. It is an attempt to mimic some of the biological processes of a real cell. The challenge is how to explore the origins of life from the biosynthesis of living systems in the laboratory.

## 5 Non-Coding RNA for Cellular Functions

One of the most important discoveries of the last few years has been the identification of small, non-protein-coding RNAs (ncRNAs) that act as integral regulatory components of cellular networks (Storz, 2002). ncRNAs serve an astonishing variety of functions and thus play important roles in many intracellular processes, from transcriptional regulation, gene silencing, chromosomal replication, through RNA processing and modification, mRNA stability and translation, to protein degradation, and translocation, etc. In synthetic biology, ncRNAs have been shown to have advantages in circuit engineering (Callura et al., 2010). Orthogonally acting ncRNA regulators that work independently in the same cell have been used to engineer higher-order regulatory functions (Lucks et al., 2011). Systematic identification and characterization of ncRNAs in genomes has become one of the most exciting challenges in cellular and development biology.

## 6 Compatibility of Genetic Elements and Cellular Chassis

Biological chassis allows heterologous genetic modules to be implanted and tested like what may happen to its engineering counterparts. However, unlike parts used in engineering systems, biological components are usually non-orthogonal, and will interact with genes, proteins, and metabolites of the chassis. The adaptation of the implanted modules to the intracellular network “environments” and the compatibility of the biological chassis with the genetic parts assembled remain as the core of current synthetic biology research.

## Conclusion

Despite of promising progresses in engineering biological systems, synthetic biology is still in an immature development phase. There exist great challenges and opportunities in synthetic biology applications for various life science and biotechnological purposes. Synergistic efforts from a diverse mix of multi-disciplinary teams of biologists, engineers, mathematicians, philosophers, computer experts, students, etc., are needed to make synthetic biology breakthroughs in areas such as medicine, pharmacology, agriculture, national security, the environment, and the production of bioenergy/biofuels, at the same time considering the policy implications, ethical, and risk management issues, for our society.
